# A data mining approach for classifying DNA repair genes into ageing-related or non-ageing-related

**DOI:** 10.1186/1471-2164-12-27

**Published:** 2011-01-12

**Authors:** Alex A Freitas, Olga Vasieva, João Pedro de Magalhães

**Affiliations:** 1 Integrative Genomics of Ageing Group, Institute of Integrative Biology, University of Liverpool, Biosciences Building, Crown Street, Liverpool, L69 7ZB, UK; 2 School of Computing and Centre for BioMedical Informatics, University of Kent, Canterbury, CT2 7NF, UK; 3 Institute of Integrative Biology, University of Liverpool, Biosciences Building, Crown Street, Liverpool, L69 7ZB, UK

## Abstract

**Background:**

The ageing of the worldwide population means there is a growing need for research on the biology of ageing. DNA damage is likely a key contributor to the ageing process and elucidating the role of different DNA repair systems in ageing is of great interest. In this paper we propose a data mining approach, based on classification methods (decision trees and Naive Bayes), for analysing data about human DNA repair genes. The goal is to build classification models that allow us to discriminate between ageing-related and non-ageing-related DNA repair genes, in order to better understand their different properties.

**Results:**

The main patterns discovered by the classification methods are as follows: (a) the number of protein-protein interactions was a predictor of DNA repair proteins being ageing-related; (b) the use of predictor attributes based on protein-protein interactions considerably increased predictive accuracy of attributes based on Gene Ontology (GO) annotations; (c) GO terms related to "response to stimulus" seem reasonably good predictors of ageing-relatedness for DNA repair genes; (d) interaction with the XRCC5 (Ku80) protein is a strong predictor of ageing-relatedness for DNA repair genes; and (e) DNA repair genes with a high expression in T lymphocytes are more likely to be ageing-related.

**Conclusions:**

The above patterns are broadly integrated in an analysis discussing relations between Ku, the non-homologous end joining DNA repair pathway, ageing and lymphocyte development. These patterns and their analysis support non-homologous end joining double strand break repair as central to the ageing-relatedness of DNA repair genes. Our work also showcases the use of protein interaction partners to improve accuracy in data mining methods and our approach could be applied to other ageing-related pathways.

## Background

Ageing is a widespread biological process with a growing impact on medicine and society, but its fundamental causes are still to a large extent unknown. This is particularly true about human ageing. Although there has been a significant progress in identifying a large number of ageing-related genes [1], [2], research in this area up to now has focused mainly on simpler model organisms. A major problem with research on ageing in humans is that it is much more difficult to do experiments, both for obvious ethical reasons and the long experimental time required, and thus shorter-lived models are usually preferred. This problem creates both a need and an opportunity to deploy bioinformatics methods to research human ageing. There is already a large amount of data on this topic in publically available gene/protein databases, but such data is still under-explored. This work aims at filling this gap, proposing a data mining approach to the analysis of ageing-related human genes.

We focused our analysis on DNA repair genes, since this is one of the major types of genes often associated with ageing [3], [4], [5]. There are more than 150 human DNA repair genes [6], [7], which are crucial for maintaining genomic integrity. DNA is constantly being damaged by numerous factors - e.g., at physiological temperatures and pH, cytosine bases spontaneously deaminate to uracil bases 100 to 500 times per day in a typical mammalian cell [8]. Although mammalian cells have a remarkable DNA repair capacity, no repair system is perfect, and DNA damage tends to accumulate with time. This accumulation is believed to be a major cause of human ageing [4], [9], [10].

There are many DNA repair genes whose defect or absence has been shown to be associated with ageing in several species, including mice and humans [5], [11]. However, not all defects in DNA repair genes result in an accelerated ageing phenotype. For instance, defects in some of those genes can be so serious that the organism dies at an embryonic or perinatal stage, whilst defects in other DNA repair genes can lead to cancer. In any case there are a number of DNA repair genes that, when defective or absent in an organism, lead to "accelerated ageing" in that organism, the so-called "progeroid syndromes". Arguably the most well-known example of such progeroid syndromes is Werner's syndrome, caused by a mutation in the *WRN *gene, which encodes a DNA helicase and exonuclease protein. Patients with Werner's syndrome start to show signs of premature ageing much earlier than usual, when they are young adults, and die at an average age of 47 [12].

Although many DNA repair genes have been associated with mammalian ageing via mutations in patients or genetic manipulations in mice, to the best of our knowledge there has been no systematic study of what are the differences between ageing-related and non-ageing related DNA repair genes. The goal of this work is to study these differences in a systematic way, focusing on human DNA repair genes, using classification methods from the area of data mining or machine learning [13]. Classification is a type of supervised learning task where each data instance (also called "an example" in data mining terminology) in the dataset consists of two parts, a set of predictor attributes and a special class attribute. A classification algorithm builds a classification model that, based on the values of the predictor attributes for a given data instance, predicts what is the class value for that instance.

This paper reports the results of applying two types of classification algorithms - decision tree induction and Naive Bayes - to a number of different datasets of DNA repair genes created specifically for this research. The resulting classification models are then interpreted in light of biological knowledge. To the best of our knowledge, this research is the first application of data mining-based classification methods to the problem of systematically determining gene properties that discriminate between ageing-related and non-ageing related DNA repair genes and, considering that the data mining methodology proposed here is generic, it could have other applications in ageing research.

## Results

We created datasets where each data instance represents a DNA repair gene. Each data instance (DNA repair gene) belongs to a class, either ageing-related or non-ageing related, and is characterized by a set of predictor attributes which encompass properties related to its DNA repair pathway, Gene Ontology (GO) term annotations, protein-protein interaction information, etc - see Methods. Each dataset is divided into a training dataset and a testing dataset. Classification algorithms were used to build, from the training set, classification models that predict the class of an instance (DNA repair gene) based on the values of predictor attributes. Each classification model is then applied to the test set, consisting of data instances unseen during training, in order to measure the predictive accuracy (generalization ability) of that model.

We report computational results for two types of classification algorithms, namely J48 and Naive Bayes (see Methods). Both are implemented in the WEKA data mining tool [13], which was used in our experiments. We report results for two types of datasets: (a) 15 datasets (variations of each other) that use multiple types of predictor attributes but not gene expression attributes; (b) a dataset including only gene expression attributes (extracted from Genevestigator). The datasets created in this work have, depending on the criteria used (see Methods), between 135 and 148 data instances, out of which 33 represent ageing-related DNA repair genes and the remaining represent non-ageing-related DNA repair genes. All the datasets created and used in this research are available on the web http://genomics.senescence.info/genes/DNA_repair.html.

### Results for datasets with multiple types of attributes but not gene expression attributes

For each classification algorithm, we report its predictive accuracy when mining different datasets produced with three different values of the parameter "GO term occurrence threshold", which specifies the minimum number of occurrences in the dataset for a GO term to be used as an attribute (see Methods), namely 3, 7 and 11. The value 3 is a conservative, low value, which avoids the use of rare GO terms with very little statistical support and virtually no generalisation power. Increasing the threshold value has two opposite effects. On one hand, although the classification algorithms are given a smaller set of attributes (because fewer GO terms satisfy the occurrence threshold), this smaller set has the advantage of including only GO terms with a larger occurrence in the dataset, for which the probabilities or related statistics computed by the classification algorithms are more reliable than statistics computed for GO terms with fewer occurrences in the dataset. On the other hand, if the threshold value is increased too much, GO terms which are relatively rare but still have some predictive power would be lost, which could lead to a decrease in classification accuracy. Hence, experiments with different values of this parameter allow us to study the aforementioned trade-off between statistical reliability and availability of relevant attributes for prediction.

We also did experiments with five different datasets produced by varying the set of protein-protein interaction (PPI)-related attributes. More precisely, dataset D1 does not contain any PPI-related attribute. Dataset D2 contains the numerical #partners (number of interaction partners) attribute, but not the binary attributes indicating whether or not the current protein interacts with a given protein - referred to as BPI (Binary Protein Interaction) attributes for short. Datasets D3, D4 and D5 contain both the #partners attribute and 10, 20 or 30 (respectively) BPI attributes. All datasets included the DNA repair pathway and the *K*_*a*_/*K*_*i *_ratio (which measures molecular evolution rates) attributes, as described in the Methods.

Table 1 shows the predictive accuracy (measured by the Area Under the ROC curve (AUC) value - see Methods) obtained by the J48 decision tree induction algorithm. The AUC value can vary from 0 to 100%; where 50% corresponds to random predictions and 100% corresponds to all correct predictions. Several relevant remarks can be made about this table. First, overall, using BPI attributes considerably increases predictive accuracy. For each of the three values of the GO term occurrence threshold, the AUC value obtained using BPI attributes is considerably greater than the AUC value obtained without that type of attribute. This tendency is particularly clear in the column for the threshold value of 3, where the AUC value for dataset D1 (with no BPI attribute) was 63% and the AUC values for datasets D3-D5 varied from 72.3% to 80%.

**Table 1:** Area under ROC curve (AUC, in %) for J48 algorithm, for different datasets and different values of the GO term occurrence threshold (t)

Dataset Id	PPI-related attributes	t = 3 (301 GO terms)	t = 7 (157 GO terms)	t = 11 (101 GO terms)
D1	none	63.0	68.0	65.3

D2	#partners	66.1	63.3	59.6

D3	#partners + 10 BPI attr's	72.3	74.2	75.4

D4	#partners + 20 BPI attr's	**80.0**	73.5	74.6

D5	#partners + 30 BPI attr's	79.2	67.7	77.5

Concerning the effect of different values of the GO term occurrence threshold in the predictive accuracy of J48, increasing the value of that threshold to 7 or 11 had mixed effects. In particular, those increased threshold values led to higher AUC values in datasets D1 and D3, but lower values in datasets D2, D4 and D5, by comparison with the AUC values associated with the original threshold value of 3. Overall, taking into account all datasets, the best results are achieved with the GO term occurrence threshold set to 3, and the best two results in the entire table are achieved for datasets D4 and D5 with the GO term occurrence threshold value of 3, corresponding to AUC values of 80.0% (boldfaced in Table 1) and 79.2%, respectively. In any case, the predictive performance of J48 was more sensitive to variations in the types of predictor attributes used in the dataset than to variations in the value of the GO term occurrence threshold.

The results for Naive Bayes are reported in Table 2. In general, for all three values of the GO term occurrence threshold, Naive Bayes' AUC value increased monotonically, from the first row (D1) to the last row (D5), with an increase in the number of PPI-related (#partners and BPI) attributes. In the case of Naive Bayes, increasing the value of the GO term occurrence threshold to 7 or 11 led to somewhat lower AUC values in four datasets (D1-D4), by comparison with the AUC values associated with the original threshold value of 3. However the highest AUC value in Table 2 was achieved with that threshold set to 11, for dataset D5 (AUC = 82.6%). In summary, varying the value of the GO term occurrence threshold had little effect on the predictive accuracy of Naive Bayes, which is more affected by the types of predictor attributes used in the dataset.

**Table 2:** Area under ROC curve (AUC, in %) for Naive Bayes, for different datasets and different values of the GO term occurrence threshold (t)

Dataset Id	PPI-related attributes	t = 3 (301 GO terms)	t = 7 (157 GO terms)	t = 11 (101 GO terms)
D1	none	75.9	74.9	71.9

D2	#partners	76.0	75.3	74.0

D3	#partners + 10 BPI attr's	78.3	77.1	76.6

D4	#partners + 20 BPI attr's	80.5	80.1	79.4

D5	#partners + 30 BPI attr's	80.7	80.2	**82.6**

To summarise the distribution of the most relevant attributes selected by J48, Table 3 shows how many times each attribute was selected to be at the root node (the most important node) of the decision tree. In Table 3, the four attributes with binary values (two GO terms, XRCC5_interaction and WRN_interaction) have their "yes" value associated with ageing - i.e., if the value of that attribute is "yes" for a given DNA repair gene, that gene is predicted to be associated with ageing. In the case of the numerical #partners attribute, J48 chose the threshold of 15 as the best value to discriminate among the two classes, so that in general values of that attribute greater than 15 partners tend to be more associated with ageing. This result is consistent with other investigations showing that ageing-related proteins tend to have a higher number of interaction partners than non-ageing-related proteins [14], [15], [16].

**Table 3:** Frequency of occurrence as root node in decision tree built by J48

Attribute	Frequency
WRN_interaction	6 (out of 6)

XRCC5_interaction	2 (out of 9)

#partners	2 (out of 12)

GO:0009719 (response to endogenous stimulus)	3 (out of 5)

GO:0042221 (response to chemical stimulus)	2 (out of 15)

### Results for datasets with gene expression attributes only

We applied the J48 and Naive Bayes classification algorithms to the dataset of DNA repair gene expression values across human tissues extracted from Genevestigator (see Methods). The AUC values obtained by these algorithms in this dataset were 51.1% and 52.1%, respectively. These values are much lower than the AUC values for the datasets with multiple types of attributes but not gene expression attributes, and they are just slightly higher than the AUC value expected from random predictions, which is 50%. Hence, the entire classification models built from this data are not reliable. However, analysing the decision tree built by J48, we found a path in the decision tree corresponding to a classification rule which is a good predictor of ageing-related DNA repair genes. This rule, which is a modular component of the classification model that can be interpreted independent from the rest of the tree, is as follows:

IF (T-lymphocyte > 6265.926) AND (tongue_squamous_cell ≤ 11127.391)

THEN class = aging_related_DNA_repair

The actual gene expression values in the IF part of the rule are not easily interpretable, but the rule can be broadly interpreted as: IF a DNA repair gene has a high expression (by comparison with other DNA repair genes) in T-lymphocytes and does not have a very high expression in tongue squamous cells, then the gene is predicted to be ageing-related. The main condition in this rule - i.e. the condition which is better at predicting the ageing-related class - is a high expression in T-lymphocytes; the other condition was added to the rule by J48 only to make the rule more consistent with respect to the underlying dataset. There are 5 DNA repair genes satisfying the IF part of this rule, and all of them have the class predicted in the THEN part - i.e. this rule has 5 "true positives" and no "false positive". This pattern is highly statistically significant (p < 0.001, with the null hypothesis of binomial distribution with probability of occurrence of the ageing-related class = 0.22 (relative frequency of this class in the dataset)). The DNA repair genes covered by this rule are: APEX1, ERCC5, RPA1, XRCC5 and XRCC6.

**Figure 1:**
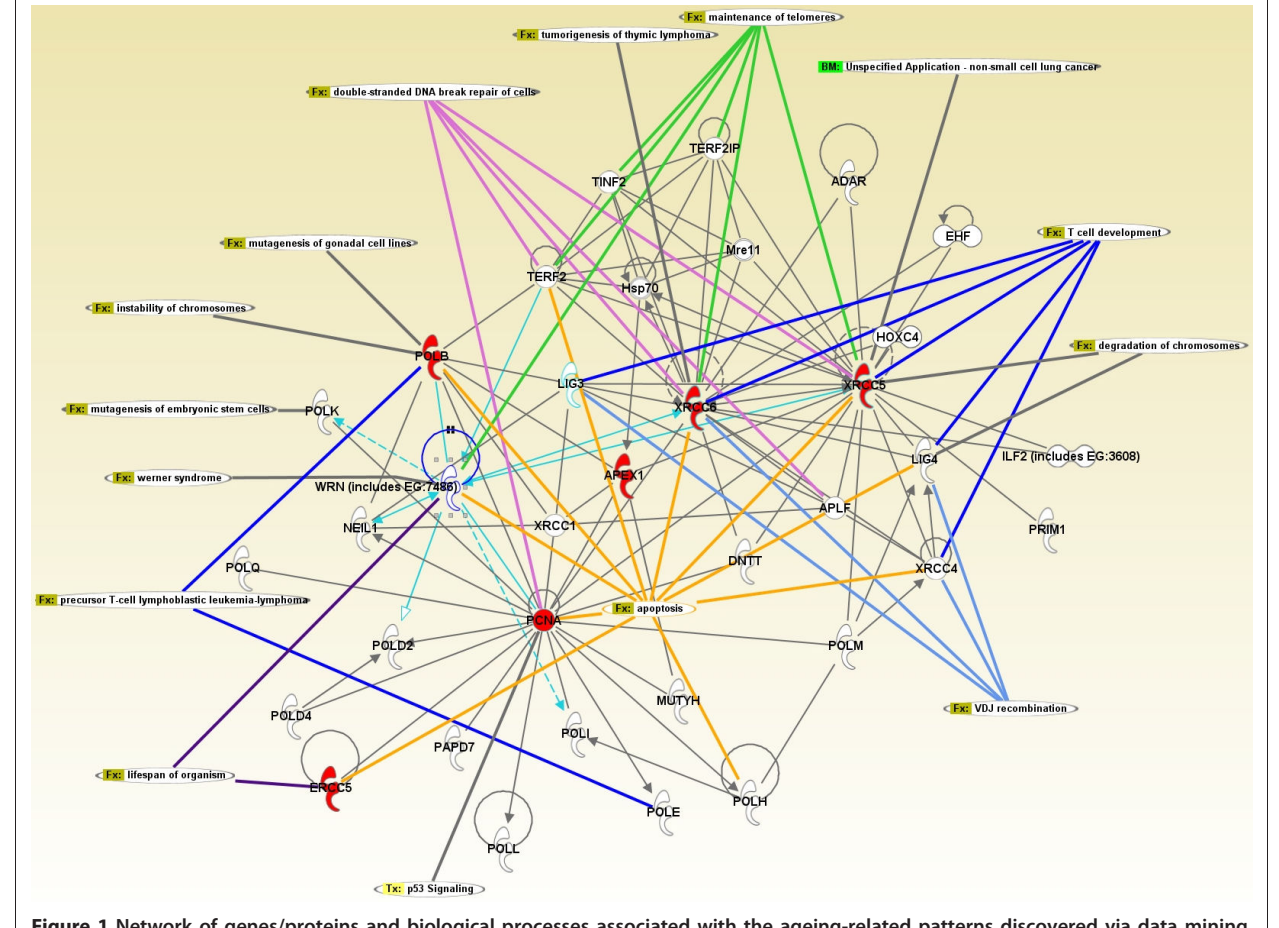
**Network of genes/proteins and biological processes associated with the ageing-related patterns discovered via data mining**. Pink links connect proteins to the process of double-strand DNA break repair, green links connect proteins to the process of telomere maintenance, dark blue to T cell development, light blue to V(D)J recombination, and yellow to apoptosis. Figure generated through the use of Ingenuity Pathways Analysis.

We therefore used the Ingenuity Pathways Analysis http://www.ingenuity.com to define crosstalk between genes/proteins involved in the patterns that were discovered via data mining. The network automatically generated by Ingenuity is mainly composed of known protein-protein interactions (PPIs). We also imposed known (and stored in Ingenuity database) links to physiological processes and diseases on the reconstructed network, shown in Figure 1. As can be observed in Figure 1, XRCC5 and XRCC6 (two of the genes whose expression was observed to be high in T lymphocytes) have particularly important roles in this network. More precisely, XRCC6 is directly connected to all 5 aforementioned processes, whilst XRCC5 is directly connected to 4 of those processes (the only exception being V(D)J recombination).

## Discussion

### Results for the datasets with multiple types of attributes but not gene expression attributes

One of the advantages of a classification model in the form of a decision tree is that it shows the relevant attributes selected by the algorithm (the attributes labelling the internal nodes of the tree) in a simple and intuitive hierarchical way, where attributes at the top of the tree are more relevant than attributes at the bottom. The attribute at the root node, in particular, is considered the most relevant attribute for classification.

As can be seen in Table 3, only two GO terms were chosen to be root nodes of a decision tree. Both terms refer to response to stimulus, but in both cases the definition of the kind of stimulus in question seems somewhat broad. A simple IF-THEN classification rule that can be extracted from the decision trees, using only the GO term "response to endogenous stimulus" at the root node to make a class prediction, is the rule:

IF GO:0009719 (response to endogenous stimulus) = yes

THEN class is aging-related DNA repair gene

There are four DNA repair genes satisfying the IF part of this rule, and all of them belong to the predicted class. This rule is statistically significant (p < 0.01, with the null hypothesis of binomial distribution with probability of occurrence of the ageing-related class = 0.23 (relative frequency of this class in the dataset)).

Another rule that can be extracted from the decision trees is:

IF GO:0050896 (response to stimulus) = yes

AND GO:0048518 (positive regulation of biological process) = yes

AND #partners > 15

THEN class is aging-related DNA repair gene

There are 10 DNA repair genes satisfying the IF part of this rule, and all of them belong to its predicted class. This rule is highly statistically significant (p < 0.001, with the above null hypothesis).

The above two rules indicate that "response to stimulus" is in general a good predictor of ageing-relatedness for DNA repair genes. It is worth mentioning that the GO term "response to external stimulus" was one of the GO terms overrepresented in an ageing-related interaction network of a very different type of gene/protein, namely extracellular proteins [17]. This suggests that the relevance of "response to stimulus" for predicting ageing-relatedness is not limited to DNA repair genes.

Interestingly, the BPI (binary protein interaction) attributes were chosen to be decision tree root nodes more often than the GO term attributes, even though there are much fewer BPI attributes than GO term attributes in the datasets. In particular, the WRN_interaction attribute (which takes the value "yes" or "no" depending on whether or not the current DNA repair protein interacts with the WRN protein) was chosen as the root attribute in 6 out of 6 of the experiments where it was input in the dataset used. The WRN protein is associated with Werner's syndrome, considered the progeroid syndrome that most presents characteristics of accelerated ageing [10], [12], and the WRN protein is a hub (a node with a large number of neighbours) in ageing-related protein interaction networks [18], [19]. It should be noted, however, that there is a bias in this result, since the WRN protein and its interaction partners tend to be more studied in the context of ageing than other types of proteins. In fact, in our datasets the WRN protein has 14 DNA repair interaction partners, all of them belonging to the ageing-related class. Therefore, although this pattern is statistically significant, it is not surprising given the biases in the datasets. Binary protein interaction data is susceptible to biases related to certain proteins being more studied than others, though apart from WRN we are confident that our results reflect real enrichments.

A more interesting pattern is that interaction with the XRCC5 (X-ray repair complementing defective repair in Chinese hamster cells 5, also called Ku 80) protein is a strong predictor of the ageing-related class. More precisely, XRCC5 was selected 2 times as the root node of a decision tree built by J48; and, out of XRCC5's 11 DNA repair interaction partners, 10 are ageing-related DNA repair proteins. This pattern is also highly statistically significant (again, p < 0.001, with the above null hypothesis).

XRCC5 is a DNA helicase involved in double-strand-break repair. Ku is a heterodimer composed of Ku70 and Ku80 subunits. When a double-strand break occurs, Ku binds to DNA ends and recruits DNA-dependent protein kinase subunit, which is believed to phosphorylate and activate downstream targets in the non-homologous end joining (NHEJ) DNA repair pathway [20]. Ku80 ^-/- ^mice, which are defective in double-strand DNA break repair via the NHEJ pathway, exhibit multiple symptoms of accelerated ageing [5], [21].

### Results for the dataset with gene expression attributes only

In this type of dataset, the main pattern discovered was that DNA repair genes having a high expression in T lymphocytes tend to be ageing-related genes, and among the genes satisfying this pattern are XRCC5 and XRCC6. In addition, as discussed earlier, interaction with XRCC5 and WRN are strong predictors of ageing-relatedness for DNA repair genes. Integrating these patterns, it is interesting to note that WRN, XRCC5, XRCC6, and T lymphocytes are all related to Non-Homologous End Joining (NHEJ), an important pathway for the repair of double-strand DNA breaks [22]. This process is required for proper telomere maintenance, and NHEJ is also required for joining hairpin-capped double-strand breaks induced during V(D)J recombination, the process that generates diversity in B-cell and T-cell receptors in the vertebrate immune system [23], [24].

Relations of DNA double-strand break frequency with telomere maintenance and ageing have been reported [9], [25], and the link between ageing and autoimmunity is also striking. Increased autoimmunity is observed in Down syndrome, which is also characterized by accelerated ageing [26], [27]. Autoimmunity was also shown to be associated with the normal ageing process [28], [29] and many ageing-related diseases (such as cancer) have an autoimmune component in their etiology.

Human T lymphocytes represent a well-characterized example of a cell type which retains the ability to up-regulate telomerase as part of their response to a proliferative stimulus [30], and can be long-lived. Defects in NHEJ that would affect other somatic cells and increase their ageing rate will have less effect in the lifespan of T-lymphocytes, but they could target their autoimmune properties. Stressing this view we may mention two facts. First, XRCC6, one of the ageing-related genes in our dataset with a high expression in T lymphocytes, is not only a DNA repair protein but also a thyroid 70 kDa autoantigen. Second, APEX1 (another gene in our dataset with a high expression in T lymphocytes) is an element in a pathway of response of a target cell to granzyme A, a protease released by cytotoxic T cells and natural killer cells. Cleaving the oxidative repair protein Ape1 enhances cell death mediated by granzyme A [30]. It is tempting to speculate that these results support connections between a faulty DNA repair system and the immune system as one of the factors influencing ageing.

## Conclusions

We have proposed the use of classification algorithms from the area of data mining (or machine learning) to analyse data about DNA repair genes associated or not with ageing. For this we created datasets specifically for our data mining purposes, integrating data from several biological databases and websites. In total 16 datasets were created, involving distinct combinations of different types of predictor attributes, namely the type of DNA repair pathway, a measure of a gene's rate of evolutionary change, a number of attributes referring to biological process GO terms (varying from 101 to 301 terms), an attribute for the number of proteins interacting with a gene's protein product, a number of attributes referring to interaction with specific proteins (varying from 0 to 30 protein interactors), and attributes involving gene expression data. We then built classification models that predict whether a given DNA repair gene is ageing-related or not. Broadly speaking, the predictor attributes used in this work were better at predicting the ageing-related class than the non-ageing-related class. This may be due to the larger diversity of genes/proteins in the latter class.

In most of the datasets, both J48 and Naive Bayes built classification models with reasonably good predictive accuracy. The two predictor attributes representing the type of DNA repair pathway and a measure of a gene's rate of evolutionary change had little predictive power. A set of predictor attributes based on a large number of GO terms (from about 100 to about 300 attributes depending on the dataset) had some predictive power. In particular, GO terms related to "response to stimulus" turned out to be reasonably good predictors of ageing-relatedness for DNA repair genes. However, the predictive accuracies achieved by both algorithms were in general considerably increased when the dataset contained not only GO term attributes but also a relatively small set of protein-protein interaction (PPI)-related attributes (from 10 to 30 attributes). An analysis of the decision trees built by J48 for different datasets revealed that, in datasets where both GO terms and PPI-related attributes are used, the latter tended to be chosen more often to label the root node of the trees, suggesting their greater relevance for the classification of DNA repair genes.

One of the patterns discovered using protein-protein interaction attributes is that, if a DNA repair gene's protein product interacts with XRCC5 (Ku80), that gene is likely to be ageing-related. Another pattern discovered using gene expression data is that DNA repair genes with a high expression in T lymphocytes tend to be ageing-related. Among the genes satisfying this latter pattern are XRCC5 and XRCC6, genes involved in non-homologous end-joining, an important DNA repair pathway for double-strand break repair and also involved in telomere maintenance and the joining of hairpin-capped double-strand breaks induced during V(D)J recombination - the process that generates diversity in B-cell and T-cell receptors in the vertebrate immune system. These patterns and processes have been further integrated in our analysis by using the Ingenuity Pathways Analysis to define crosstalk between genes/proteins involved in the aforementioned patterns and processes.

Future research will consist of building classification models based on other types of predictor attributes, e.g. involving information about the domains composing the DNA repair proteins being investigated and information about gene essentially [31]. In the future it could also be interesting to apply this proposed methodology to the analysis of other types of genes associated with ageing too, e.g. perhaps to discriminate between ageing-related and non-ageing-related genes associated with oxidative stress.

## Methods

### Creating datasets with multiple types of predictor attributes

Each data instance represents a DNA repair gene and consists of two parts, a set of predictor attributes and a class. The class of a data instance can either be: ageing-related DNA repair or non-ageing-related DNA repair gene. To perform these class assignments, first, a set of DNA repair gene names was obtained from http://sciencepark.mdanderson.org/labs/wood/DNA_Repair_Genes.html (hereafter called "Wood's web site" for simplicity) [6], [7]. The genes in that set were then divided into two classes. The positive (ageing-related) class consists of the genes included in the GenAge database of ageing-related genes http://genomics.senescence.info/genes/[2]. The negative (non-ageing-related) class consists of the genes that are not included in GenAge.

Each data instance (in either class) was then represented by a set of predictor attributes. Several types of predictor attributes - representing different types of properties of the genes in the dataset - have been created, as follows.

#### Creating the predictor attribute type of DNA repair

This attribute represents the main type of DNA repair process in which a gene is involved, using information derived from Wood's web site. This attribute has 12 possible values, namely: base excision repair, mismatch repair, nucleotide excision repair, homologous recombination, non-homologous end joining, other types of DNA repair, DNA polymerases (catalytic subunits), editing and processing nucleases, Rad6 pathway, disease, other genes with known or suspect DNA repair function, other conserved DNA damage response genes. For a definition of these types of DNA repair, see [6], [7], [8].

#### Creating a predictor attribute measuring the rate of evolutionary change (K_a_/K_i _ratio)

This attribute is essentially a measure of the rate of evolutionary change of orthologous ageing-related genes in human and chimpanzees, called the *K*_*a*_/*K*_*i *_ratio. The values of this attribute used in our dataset were taken from [32].

#### Creating a set of predictor attributes representing GO terms

The Gene Ontology categorizes gene/protein functions into three separate "namespaces": biological process, molecular function and cellular component [33]. We used as predictor attributes only biological process (BP) GO terms, which are more easily interpretable as attributes for predicting whether a DNA repair gene is ageing related or not.

It is important to note that, in most gene/protein databases with GO term annotations, only the most specific GO terms known for a gene are explicitly included in the database. Ancestors of those specific terms, representing more generic functions, are not normally explicitly included in the database record for that gene. However, the semantics of the Gene Ontology specifies a hierarchical relationship between terms, so that if a gene has a certain biological process function associated with it, this means the gene also has all its "ancestor (more generic) functions (terms)" in the GO hierarchy. If information about those more generic terms is not included in the dataset, the algorithm could easily compute wrong probabilities or related statistics in the data. To avoid this problem the hierarchical relationship among GO terms was taken into account when creating the datasets. First, for each DNA repair gene, we obtained the list of all the most specific GO terms annotated for that gene in the UniProt database http://www.uniprot.org. Next, for each DNA repair gene, we extended its list of specific BP GO terms with the set of all GO terms that are ancestors of those specific terms according to the "is a" relationship of the GO, using information from the Gene Ontology web site http://www.geneontology.org.

**Figure 2:**
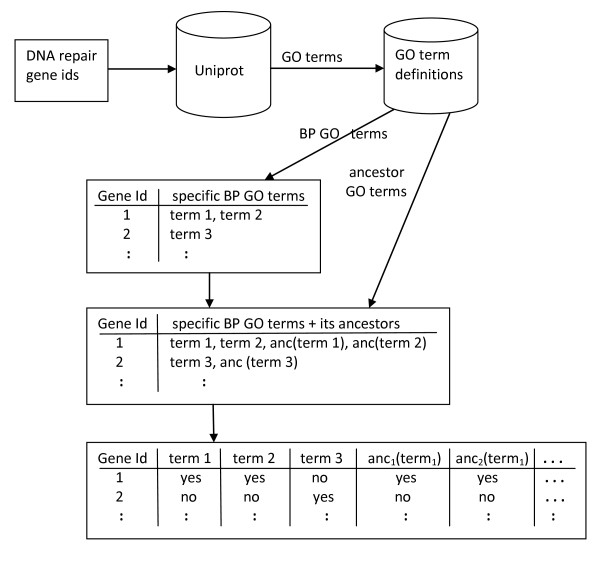
**Summary of the procedure for creating a set of predictor attributes involving GO terms**. First, a list of gene IDs is used to download from UniProt the specific GO terms annotated for each gene. Next, information about GO term definitions is used to select only the biological process (BP) terms for each gene, and then to find the ancestors of those terms in the GO hierarchy. (The notation "anc(term_1_)" denotes the set of all ancestors of term_1, _"anc_1_(term_1_)" denotes the first ancestor of term 1, etc.) After adding those ancestor GO terms to the list of GO terms per gene, the dataset is transformed into a format having a fixed-length list of binary attributes (representing GO terms) for each gene, where each attribute value indicates whether or not the gene is annotated with the corresponding GO term.

Since most classification algorithms assume that each data instance (gene/protein) has the same number of attributes, all data instances were represented by a fixed set of attributes representing a binary value ("yes" or "no")" for each GO term. These steps are summarized in Figure 2.

Many GO terms are associated with just one or two genes in the dataset, and therefore they correspond to predictor attributes with no or very low predictive power. Hence, GO terms whose value "yes" has a frequency of occurrence smaller than a predefined threshold - the "GO term occurrence threshold" - are removed from the dataset.

#### Creating a set of attributes representing protein-protein interaction information

Information about protein-protein interaction (PPI) was obtained from the HPRD (Human Protein Reference Database) - http://www.hprd.org/. HPRD was chosen because it focuses specifically on human proteins and because the curation of its data is of high quality [34]. PPI-related attributes were created as follows. First, we downloaded the dataset of PPIs from HPRD (Release 8) and selected a subset of those interactions satisfying two conditions: (a) at least one of the two proteins in the interacting pair is a DNA repair protein in our dataset; (b) the type(s) of evidence for the interaction includes *in vitro *or *in vivo *experiments - i.e. interactions for which the only type of evidence is high-throughput experiments were not selected, since this is considered a weaker type of evidence.

Two types of predictor attributes derived from those PPIs were used. The first type of PPI attribute is the number of interaction partners (#partners) for a given protein. The second type of PPI-related attribute involves a set of binary attributes, as follows. For each data instance (gene) in our dataset, each attribute takes on the value "yes" or "no" to indicate whether or not (respectively) that gene's protein product interacts with the protein represented by that attribute. The set of PPIs selected from HPRD involved 656 interacting proteins, and it was not practical to create one predictor attribute for each of those proteins. Hence, we created a set of *N *binary attributes referring to the *N *most frequent proteins in the PPIs selected from HPRD, where *N *is a parameter used in the creation of the dataset.

#### Removing duplicated data instances

After creating the above-described set of predictor attributes, it often happens that a few data instances - representing DNA repair genes which are similar to each other - contain the same values for all attributes. Hence, to eliminate data redundancy from a data mining perspective, as a final step in the creation of the datasets, we detect and remove duplicate instances in the data. The actual number of duplicates depends on the specific attributes included in each created dataset. More precisely, the datasets described so far have between 135 and 140 data instances, out of which 33 represent ageing-related DNA repair genes and the remaining represent non-ageing-related DNA repair genes.

### Creating a dataset with gene expression attributes only

Genevestigator is a system for investigating gene expression and gene regulation https://www.genevestigator.com/[35]. We used the system's Anatomy tool, which reports how strongly a gene is expressed in different anatomical categories, including tissues and cells.

To create attributes representing gene expression levels reported by the anatomy tool, we used the aforementioned lists of ageing-related and non-ageing-related DNA repair genes and searched for expression profiles pre-selected by the annotation adult human tissue in the Genevestigator's anatomy array collection. For each anatomy category, Genevestigator displays the average expression value calculated from all arrays in the focused array selection that are annotated as belonging to this category.

Note that Genevestigator contains expression data from multiple types of microarrays, e.g., different generations of Affymetrix GeneChips. On these arrays, individual genes are sometimes represented by different sets of probes, which are not mixed. To get an average of all the existing data, we used all probes corresponding to one gene in our analysis, by computing the arithmetic average of all gene expression values (one for each probe) for each gene. Hence, we created a dataset where each instance corresponds to a DNA repair gene and each column (attribute) corresponds to an anatomical category - i.e., each attribute value is the average expression level of a given gene for all probes in the corresponding anatomical category. After all data preparation steps, the created dataset has 148 data instances and 108 attributes.

### Classification algorithms

We used two types of classification algorithms in our experiments. The first type is a decision tree induction algorithm, more precisely the well-known J48 algorithm, implemented in the data mining tool WEKA [13]. J48 builds a decision tree, where each leaf node is labelled with one of the classes to be predicted (ageing-related or non-ageing-related DNA repair gene in our case) and each internal (non-leaf) node represents a test on the value of a predictor attribute labelling the node. Note that a decision tree algorithm selects only the most relevant attributes to be included in the decision tree, and many attributes may not appear in the tree at all because they are not necessarily considered relevant for class prediction by the algorithm. In addition, for the attributes which are selected to be included in the decision tree, in general, the closer to the root the attribute is, the more relevant for class prediction it is.

The Naive Bayes algorithm assigns to a data instance the class *k *that maximises the product: P(*A*_*1*_*|C*_*k*_) × P(*A*_*2*_*|C*_*k*_) × . . . P(*A*_*m*_*|C*_*k*_) × P(*C*_*k*_), where P(*A*_*i*_*|C*_*k*_) - *i *= 1,...,*m *- is the empirical conditional probability of the value of attribute *A*_*i *_in the current data instance given that the instance belongs to class *k *(i.e., the number of training data instances having that value of attribute *A*_*i *_and having class *k *divided by the number of training data instances having class *k*), *m *is the number of predictor attributes, and P(*C*_*k*_) is the empirical prior probability of class *k *(i.e. the relative frequency of class *k *in the training set). Naive Bayes makes the simplifying assumption that the attributes are independent from each other given the class. Although this simplifying assumption is not true in many cases, the algorithm still performs robustly well in practice. Also, more complex types of Bayesian classifiers, which detect dependences among attributes, would tend to lead to overfitting [13] in our small dataset.

J48 and Naive Bayes are both popular data mining algorithms that have the advantage of producing a classification model in a format that can be interpreted by biologists. This is in contrast with "black box" algorithms such as Support Vector Machines (SVMs) [36] - which tend to obtain somewhat higher predictive accuracies but have the disadvantage of producing non-interpretable classification models. In scientific discovery applications such as in this work it is important to build classification models that can be interpreted by biologists, as discussed in [37], [38].

### Measuring predictive accuracy

The performance of the classification model is measured by its predictive accuracy in data that was not used to build the model, as follows. First, the classification model is built from a subset of the data called the training set, where the algorithm knows the values of both predictor attributes and classes for the data instances. After the model is built, its predictive accuracy is then measured in a separate subset of the data, called the test set, where the algorithm knows only the values of the predictor attributes (and not classes) for data instances. So, this measure of predictive accuracy measures the generalization ability of the classification model.

In this work, predictive accuracy is measured in terms of the Area Under the ROC curve (AUC) using 10-fold cross-validation. The AUC measure is a commonly used measure of predictive accuracy in data mining and bioinformatics. In order to interpret the AUC values in the tables of results to be reported below, the main point is that the larger the AUC value, the better the predictive accuracy of the classification model - in particular, a perfect predictive model would have an AUC value of 1 (100%), whilst a model that makes predictions entirely at random would have an AUC value of 0.5 (50%). ROC analysis and the AUC measure are described in detail e.g. in [39]. 10-fold cross-validation is a very common procedure for estimating predictive accuracy. In essence, it works as follows [13]. First, the dataset is divided into 10 folds of approximately equal size. Next, the classification algorithm is run 10 times, each time with a different fold used as the test set and all the other 9 folds merged into the training set. The predictive accuracy measure (the AUC value in our case) is computed as the average value of that measure in the test set over the 10 experiments. Hence, each data instance is used exactly once in the test set and 9 times in the training set.

### Statistical significance

The statistical significance of specific attribute values predicting the ageing-related class (as found by the data mining algorithms) was measured by using a hypothesis test based on the binomial distribution, as follows. For a given attribute value or rule predicting the ageing-related class, the number of successes was the number of data instances (DNA repair genes) which have that attribute value or satisfy the IF part of the rule and belong to the ageing-related class; the number of trials was the number of data instances that have that attribute value or satisfy the IF part of the rule (regardless of their class); and the null hypothesis was represented by a binomial distribution where the probability of occurrence of the ageing-related class is the relative frequency of that class in the entire dataset.

## Authors' contributions

AAF created the datasets, ran the classification algorithms, participated in the analysis of the patterns discovered by the algorithms, and drafted most of the manuscript. OV used the Genevestigator software to obtain gene expression data, used the Ingenuity software to help to analyze the discovered patterns, and helped to draft the manuscript. JPM conceived the goal of the project, participated in the analysis of the discovered patterns, coordinated the project and helped to draft the manuscript. All authors read and approved the final manuscript.
